# Insights into the Quorum-Sensing Activity in Aeromonas hydrophila Strain M013 as Revealed by Whole-Genome Sequencing

**DOI:** 10.1128/genomeA.01372-14

**Published:** 2015-01-02

**Authors:** Wen-Si Tan, Wai-Fong Yin, Kok-Gan Chan

**Affiliations:** Division of Genetics and Molecular Biology, Institute of Biological Sciences, Faculty of Science, University of Malaya, Kuala Lumpur, Malaysia

## Abstract

Aeromonas hydrophila species can be found in warm climates and can survive in different environments. They possess the ability to communicate within their populations, which is known as quorum sensing. In this work, we present the draft genome sequence of A. hydrophila M013, a bacterium isolated from a Malaysian tropical rainforest waterfall.

## GENOME ANNOUNCEMENT

For a concerted response action to occur in a bacterial population, each bacterium must be aware of and respond to others in unison (1, 2). The term “quorum sensing” (QS) was coined to explain bacterial communication in which synchronization of small diffusible molecules in a population stimulates a series of gene expressions (3). The aquatic environment provides a reservoir for microorganisms, and the microbial contamination of water often leads to major concerns (4). Aeromonas hydrophila, commonly known as waterborne bacteria, can cause infections, such as diarrhea, in humans, where it utilizes its QS ability to coordinate and enhance adaptation to various environments (5, 6). In this study, the A. hydrophila strain M013 was isolated from the Sungai Tua waterfall. In order to further explore the genetic makeup of the QS system, whole-genome sequencing was performed.

The MasterPure DNA purification kit (Epicentre, Inc., Madison, WI, USA) was used to extract the genomic DNA, while the quality of extracted DNA was examined via a NanoDrop spectrophotometer (Thermo Scientific, Waltham, MA, USA) and a Qubit version 2.0 fluorometer (Life Technologies, Carlsbad, CA, USA). The purified DNA was subjected to whole-genome shotgun sequencing on an Illumina MiSeq personal sequencer (Illumina, Inc., San Diego, CA, USA), which generated 4,987,814 paired-end reads. The trimmed sequences (990,071 quality reads) were *de novo* assembled with CLC Genomic Workbench version 5.1 (CLC Bio, Denmark). A total of 164 contigs and an *N*_50_ of approximately 72,471 bp were generated.

The draft genome of the strain M013 isolate contained 4,967,716 bp, with an average coverage of 38-fold with a G+C content of 61%. The gene prediction was then performed with a prokaryote gene prediction algorithm by using Prodigal (version 2.60) (7), while rRNA and tRNA were predicted with RNAmmer (8) and tRNAscan SE version 1.21 (9), respectively. Subsequently, the M013 sequence was annotated with RAST (10). The analyses of the draft genomes identified 4,360 open reading frames (ORFs), 92 tRNAs, and one copy each of 5S rRNA, 16S rRNA, and 23S rRNA.

The complete ORFs of A. hydrophila strain M013 *luxI* and *luxR* homologues were predicted to be located at contig 75. This whole-genome sequence allows deeper understanding of the genetic makeup of A. hydrophila and may help in identifying the link between pathogenicity and virulence factors of this strain and its QS properties (5, 11).

### Nucleotide sequence accession numbers.

This whole-genome shotgun project has been deposited at DDBJ/EMBL/GenBank under the accession number JRWS00000000. The version described in this paper is the first version, JRWS01000000.
